# High-Precision Cavity Length Demodulation Method for Fiber-Optic Fabry–Perot Sensors Based on Dual Superluminescent Diodes

**DOI:** 10.3390/s22155898

**Published:** 2022-08-07

**Authors:** Weiguang Zhang, Jia Yu, Xiongxing Zhang, Haibin Chen, Junying Zhang, Wei Wang

**Affiliations:** School of Optoelectronic Engineering, Xi’an Technological University, Xi’an 710021, China

**Keywords:** fiber-optic sensor, Fabry–Perot cavity, interrogation, correlation

## Abstract

A high-precision cross-correlation cavity length demodulation method for fiber-optic Fabry–Perot (F–P) sensors based on two different wavelength superluminescent diodes (SLDs) was proposed. This method can solve the problem of low demodulation accuracy caused by the difficulty in identifying the maximum cross-correlation coefficient when the cavity length of the fiber-optic F–P fiber sensor is too short, or when the spectral bandwidth of the illuminating single-light source is too narrow. This demodulation method is based on the principle that the two main peaks of the two cross-correlation curves corresponding to two different spectral ranges should match, and the average value of the two calculated cavity lengths corresponding to the two matched peaks is determined as the real cavity length. The cavity length demodulation of fiber-optic F–P sensors in the range of 20–200 μm shows a maximum measurement deviation of 0.008 μm, which is significantly smaller than the demodulation result obtained with a single light source, and the standard deviation of the measurement results is only approximately 0.0005 μm, indicating the high precision and stability of a dual SLD cross-correlation demodulation method.

## 1. Introduction

The Fabry–Perot (F–P) cavity is a classical optical interference structure, which has been used in various important optical precise measurements for decades. By the fabrication of F–P cavities into a fiber-optical path, different miniature fiber-optic F–P sensors have been proposed and realized. Fiber-optic F–P sensors show anti-electromagnetic interference, are lightweight, have strong environmental adaptability, and high sensitivity due to which they have been widely used in aerospace, bridge monitoring, oil field, dam pressure monitoring, and many other fields [1,2,3]. By monitoring the cavity length variations of the fiber-optic F–P sensor, various physical parameters, such as pressure [4,5], temperature [6,7], strain [8], and vibration [9,10,11], can be measured. In particular, fiber-optic F–P sensors can survive and operate properly even in high-temperature and high-pressure environments [12,13,14] while also making the simultaneous multiple parameter measurements of temperature, pressure, and magnetic field feasible.

High-precision cavity length demodulation of fiber-optic F–P sensors is a key issue for their practical applications. Demodulation methods can be divided into two categories: intensity demodulation and phase demodulation. Intensity demodulation seeks the best linear working point and realizes a high-speed demodulation of the cavity length near this point. This method is susceptible to external factors during the measurement process, leading to weak application adaptability [15,16]. Phase demodulation commonly has a strong anti-interference capability, and many different methods have been developed, including the Fourier-transform method [16,17,18,19], peak tracking method [20,21,22,23], cross-correlation method [24,25,26,27,28,29], and so on. The Fourier-transform method is a demodulation method based on analyzing the reflection or transmission spectrum of a fiber-optic F–P sensor, which commonly requires a broadband source or a wideband tunable laser. With a wideband spectral range, the cavity length of the F–P sensor can be demodulated with high accuracy. The peak tracking method, also called the fringe counting method, determines the cavity length by determining the relative position of the peak in the reflection spectrum. The demodulation accuracy of this method is directly affected by the wavelength positioning accuracy of the spectral peak, and significant errors are easily introduced during the order-judgment processes. The cross-correlation method achieves cavity length demodulation by continuous cross-correlation between the reflection spectrum of the fiber-optic F–P sensor and the template function of a virtual F–P cavity. The length of the virtual F–P cavity varies in a range that covers the real F–P cavity. By determining the maximum value of the main peak of the cross-correlation curve, the real cavity length can be determined accurately at the nanometer or subnanometer level [26,27]. However, if the cavity length of the fiber-optic F–P sensor is relatively short or if the spectral width of the light source is not wide enough, then the main peak of the cross-correlation curve may be inaccurately positioned. Consequently, significant errors are introduced.

In general, the narrower the bandwidth the illuminating source has, the longer the lower limit of the cavity length without “mode jumping” is. Additionally, noises, background disturbances, or power spectral fluctuations of the light sources can further raise the lower limit. The spectral bandwidth of a single superluminescent diode (SLD) or amplified spontaneous emission source is typically only ~40 nm. For F–P sensors with short cavity lengths less than or a litter over 100 μm, with the use of these sources, it is often difficult to reliably extract their cavity lengths from the conventional cross-correlation algorithm because the main peak corresponding to the cavity length may not be clearly distinguishable from its neighboring peaks. A high-order harmonic-frequency cross-correlation algorithm has been proposed to solve the problem, but the lower limit of the cavity length range that can be demodulated is still rather restricted [28].

To overcome these problems of previous cross-correlation algorithms, in this study, we propose a high-precision cavity length demodulation method for fiber-optic F–P sensors based on the simultaneous use of two SLDs with different central wavelengths and a combination of two cross-correlation operations corresponding to two spectral ranges. Two cross-correlation curves between two reflected spectral signals in two different wavelength ranges and the respective template functions are calculated, and the most matching peaks are determined as the main peaks. The real cavity length is extracted as the average value of the two calculated cavity lengths corresponding to the two main peaks of the two cross-correlation curves. With the proposed method, the lower limit of the cavity length range that can be demodulated has been significantly extended. Theoretical analysis, simulations, and real demodulation experiments were conducted to verify the proposed demodulation method.

## 2. Dual SLD Cross-Correlation Demodulation Principle

### 2.1. Mathematical Model of Dual SLD Cross-Correlation Demodulation

**Figure 1:**
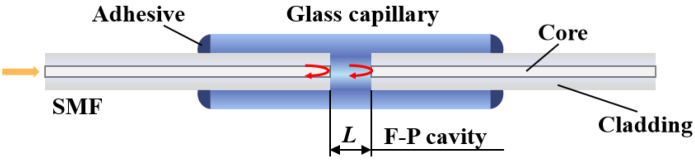
Schematic diagram of a typical fiber-optic F–P sensor.

The core part of a fiber-optic F–P sensor is an F–P cavity, which is typically composed of two parallel reflecting surfaces. Figure 1 shows the typical structure of a fiber-optic F–P sensor. Two vertically cut single-mode optical fibers (SMFs) were inserted into a glass capillary. The two separated end faces of the two SMFs form an F–P cavity.

We propose a dual SLD cross-correlation demodulation method for the high-precision demodulation of fiber-optic F–P sensors based on the principle that the main peaks of the two cross-correlation curves corresponding to two different spectral ranges should overcome the limitations of conventional demodulation methods. The method consists of the mathematical model, processing algorithm, and measurement error evaluation method, which are discussed in detail below.

**Figure 2:**
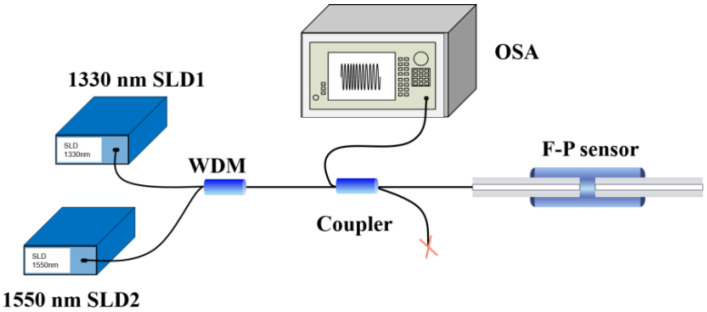
Schematic diagram of the dual SLD F–P cavity length demodulation system.

A schematic of the dual SLD cross-correlation demodulation system is shown in Figure 2. The system consists of two SLDs, a 2 × 1 wavelength division multiplexer (WDM), 2 × 2 fiber coupler, and optical spectrum analyzer (OSA). The lights with different spectral bands emitted by SLD 1 and SLD 2 are first combined by the WDM and arrives at the fiber coupler; they are then coupled to the fiber-optic F–P sensor. The reflected light-carrying cavity length information is returned to the fiber coupler and received by the OSA to obtain the reflection spectrum. The cavity length information is then extracted by a demodulating program based on cross-correlation operations in the two separated spectral ranges covered by the two SLDs.

According to the principle of F–P interference, the reflectivity $R_{r}\left(\lambda \right)$ of the fiber-optic F–P sensor in the wavelength domain can be expressed as [25]:

(1) $$R_{r}\left(\lambda \right)=\frac{R_{1}+R_{2}-2\sqrt{R_{1}R_{2}}\cos\left(\frac{4\pi nL}{\lambda }\right)}{1+R_{1}R_{2}-2\sqrt{R_{1}R_{2}}\cos\left(\frac{4\pi nL}{\lambda }\right)},$$

where $R_{1}$ and $R_{2}$ are the reflectivities of the two fiber end faces, n is the refractive index of the dielectric medium filled between the two fiber end faces, and L is the cavity length of the F–P cavity.

For a low-fineness fiber-optic F–P sensor, the reflectivity R of the end face of the fiber is much less than 1, that is, $R_{1}<<1$ and $R_{2}<<1$. Equation (1) can be approximated as

(2) $$R_{r}\left(\lambda \right)=A+B\cos\left(\frac{4\pi nL}{\lambda }\right),$$

where $A=R_{1}+R_{2}$, $B=-2\sqrt{R_{1}R_{2}}$.

The constant component A is filtered out with a high-pass filter and by normalization to obtain the reflectance spectral function.

(3) $$Y\left(\lambda \right)=\cos\left(\frac{4\pi nL}{\lambda }\right),$$

This gives out a normalized template function as

(4) $$C\left(\lambda \right)=\cos\left(\frac{4\pi nl}{\lambda }\right),$$

Suppose that the fiber-optic sensor is illuminated by two wideband sources with two different central wavelengths, we perform cross-correlation calculations between the normalized reflection spectrum function and the normalized template function in the two corresponding wavelength ranges, which gives us the following relations:

(5) $$R_{YC1}(L,l)=\int_{\lambda_{L1}}^{\lambda_{U1}}Y(\lambda )C(\lambda )d\lambda =\int_{\lambda_{L1}}^{\lambda_{U1}}\cos(\frac{4\pi nL}{\lambda })\cos(\frac{4\pi nl}{\lambda })d\lambda ,$$

(6) $$R_{YC2}(L,l)=\int_{\lambda_{L2}}^{\lambda_{U2}}Y(\lambda )C(\lambda )d\lambda =\int_{\lambda_{L2}}^{\lambda_{U2}}\cos(\frac{4\pi nL}{\lambda })\cos(\frac{4\pi nl}{\lambda })d\lambda ,$$

where $\lambda_{L1}$ and $\lambda_{U1}$, $\lambda_{L2}$, and $\lambda_{U2}$ are respectively upper and lower limits of the two spectral ranges. l is the template cavity length corresponding to the template function, and $R_{YC1}\left(L,l\right)$ and $R_{YC2}\left(L,l\right)$ are the two cross-correlation coefficients between L and l in the two spectral ranges, respectively.

In cross-correlation calculations, the variation range of l is required to cover the real cavity length L. According to Equations (5) and (6), a series of cross-correlation coefficients can be obtained, and the two cross-correlation coefficient curves can be superimposed onto the same coordinate system with the template cavity length l being the x-coordinate. There are many different peaks in the two cross-correlation curves. The separation of any two neighboring peaks on each of the cross-correlation curves is the same; however, for two different cross-correlation curves from two SLDs of different central wavelengths, the separations of two neighboring peaks are different. As a result, only when the template cavity length l=L can the two peaks coincide with each other, and with the increasing deviation of l from L, the two peaks of the same order on the two cross-correlation curves will deviate more from each other. Thus, the coincidence of the two peaks on the two different cross-correlation curves can be used as the judging criteria of the main peak determination for the real cavity length calculation.

To find the two most matched peaks, we select the difference in δ between the l positions of the peak center as the critical parameter. For the two neighboring peaks of the two cross-correlation curves, when δ is the smallest, they are determined as the two main peaks of the two cross-correlation curves, and the corresponding cavity lengths are $L_{1}$ and $L_{2}$, respectively. We can take their average value as the final measured cavity length, and we have:

(7) $$L=\frac{L_{1}+L_{2}}{2},$$

### 2.2. Processing Algorithm for Dual SLD Cross-Correlation Demodulation

**Figure 3:**
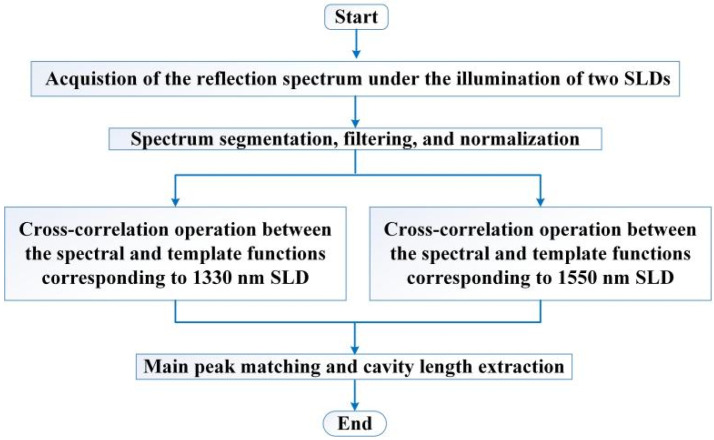
Flowchart of the processing algorithm for the dual SLDs F–P cavity length demodulation.

According to the above mathematical model, a processing algorithm for the dual SLD cross-correlation demodulation is proposed, and the corresponding flowchart is shown in Figure 3. The detailed procedures of the processing algorithm are as follows.

First, the reflection spectrum of the fiber-optic F–P sensor from two SLDs with different central wavelengths was collected by the OSA.

Second, the collected spectrum data were segmented into two parts corresponding to 1330 and 1550 nm SLDs, respectively.

Third, the direct components of the two segments of the spectrum data were filtered out using a high-pass filter and then normalized to obtain the reflection spectral functions $Y_{1}\left(\lambda_{i}\right)$ and $Y_{2}\left(\lambda_{i}\right)$ of the fiber-optic F–P sensor, where i=1,2,…,N is the pixel number of the charge-coupled device array in the OSA, and N is the total number of the pixels. The wavelength $\lambda_{i}$ is discretized because the output of the OSA is in a digital form.

Fourth, the reflection spectral functions corresponding to the two SLDs are cross-correlated with the normalized template function in the two spectral ranges according to the discretized forms of Equations (5) and (6) as below:

(8) $$R_{YC1}(L,l)=\sum_{\lambda_{L1}}^{\lambda_{U1}}Y(\lambda_{i})C(\lambda_{i})\Delta \lambda =\sum_{\lambda_{L1}}^{\lambda_{U1}}Y(\lambda_{i})\cos(\frac{4\pi nl}{\lambda_{i}})\Delta \lambda ,$$

(9) $$R_{YC2}(L,l)=\sum_{\lambda_{L2}}^{\lambda_{U2}}Y(\lambda_{i})C(\lambda_{i})\Delta \lambda =\sum_{\lambda_{L2}}^{\lambda_{U2}}Y(\lambda_{i})\cos(\frac{4\pi nl}{\lambda_{i}})\Delta \lambda ,$$

where $\Delta \lambda =\lambda_{i+1}-\lambda_{i}$ is the wavelength separation between each neighboring pixel.

Fifth, all peaks appearing in the two cross-correlation curves were positioned, and the two peaks from the two different cross-correlation curves with the minimum template cavity length difference were determined as the main peaks for the two cross-correlation curves.

Finally, the average value of the two main peaks corresponding to these template cavity lengths was determined as the measured cavity length of the fiber-optic F–P sensor.

## 3. Simulation

A simulated cavity length demodulation of fiber-optic F–P sensors with the proposed method was carried out according to a mathematical model and the processing algorithm of the dual SLD cross-correlation demodulation discussed previously.

**Figure 4:**
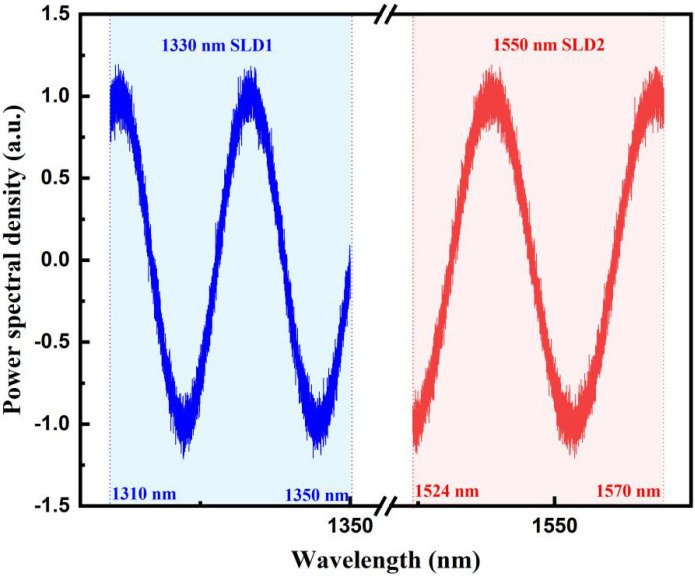
Simulated reflection spectrum of a 40 μm fiber-optic F–P sensor under the illumination of two SLDs with different central wavelengths including random noise.

First, a fiber-optic F–P sensor with a cavity length of 40 μm was simulatedly demodulated. The reflection spectrum of the sensor under the illumination of two SLDs is shown in Figure 4, in which the two SLDs were supposed to have a center wavelength of 1330 and 1550 nm and a 3-dB spectral range of 1310–1350 nm and 1524–1570 nm, respectively. These parameters were in agreement with the actual spectra of the SLDs used in the experiments.

**Figure 5:**
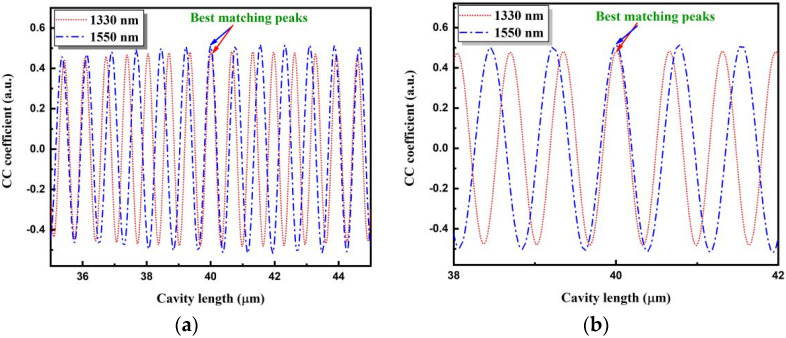
Cross-correlation curves of the 40 μm fiber-optic F–P sensor corresponding to two spectral ranges. (**a**) The template cavity length range is 35–45 μm. (**b**) The neighboring range of the best matching point is enlarged. CC: cross-correlation.

The reflection spectrum of the fiber-optic F–P sensor was first separated into two parts, which were then filtered and normalized to obtain spectral functions corresponding to the two spectral ranges. By the normalization, detrimental effects of the power fluctuation or spectral shape from the two SLDs on the cavity length extraction can be effectively shielded. Subsequently, to obtain the cross-correlation curves, cross-correlation calculations were performed between the two spectral functions using the template functions C(λ) given in Equations (5) and (6). The template cavity length covers a range of 35–45 μm, and the step size is 0.01 μm. The calculated results are shown in Figure 5a,b. Figure 5b shows a partially enlarged view of Figure 5a in the neighboring range of the best matching point.

Both cross-correlation curves oscillate with increasing or decreasing template cavity length. The cross-correlation curves exhibited many different peaks. One peak in the cross-correlation curve reaches its maximum, which is called the main peak. At this peak, the template cavity length is equal to the real cavity length. However, because the differences between the main peak and its neighboring peaks are not obvious, the main peak is difficult to distinguish. This problem can be overcome by combining two cross-correlation curves that correspond to two different spectral ranges. From Figure 5, it can be observed that the two main peaks of the two cross-correlation curves are superimposed at the same position, and resemble each other the most. Thus, from this perspective, the main peak of the cross-correlation curves can be easily determined.

The cavity lengths corresponding to the peaks of the two cross-correlation curves were compared. When the cavity length difference is minimum, the average value of the two template cavity lengths is determined as the measured length. For the 40 μm fiber-optic F–P sensor, the two peaks near the template cavity length of 40 μm were the most matched. Based on their positions, the final cavity length value was determined accurately to be 40.0035 μm, which has a small deviation of 0.0035 μm from the preset cavity length. In comparison, the cavity lengths determined by the cross-correlation operation of only one reflection spectrum of the 1310–1350 nm or 1524–1570 nm range were 40.660 and 42.322 μm, respectively. The result shows that the proposed dual-SLD cross-correlation demodulation method can improve the demodulation accuracy significantly.

**Figure 6:**
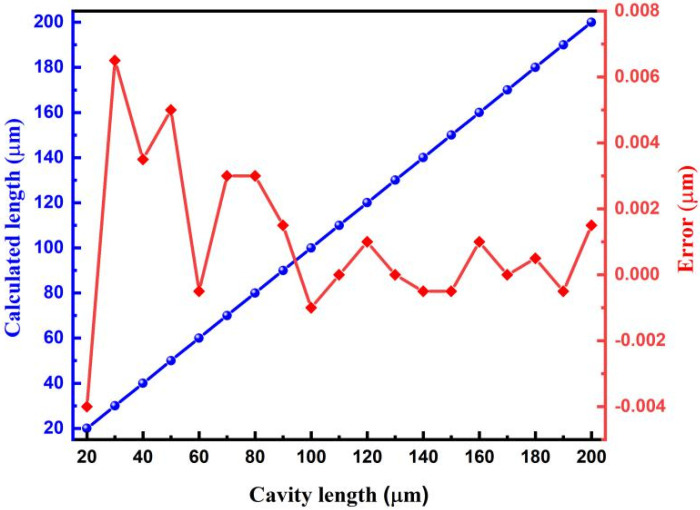
Calculated cavity lengths (**left**) and deviation errors (**right**) for fiber-optic F–P sensors in the range of 20–200 μm.

To further investigate the performance of the proposed method, 19 different fiber-optic F–P sensors with cavity lengths in the range of 20–200 μm were demodulated. The demodulation results are shown in Figure 6. An excellent linear relationship can be observed between the demodulated length and the preset cavity length, in which the maximum cavity length deviation is only approximately 0.0065 μm and the relative deviation does not exceed 0.022%.

**Figure 7:**
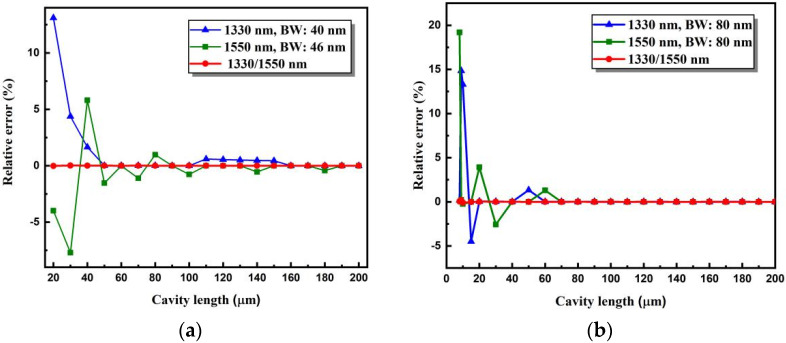
Simulated demodulation deviations of fiber-optic F–P sensors with different cavity lengths under different illuminating conditions of a single 1330 or 1550 nm SLD or two SLDs simultaneously. (**a**) The single 1330 or 1550 nm SLD has a spectral width of 40 and 46 nm, respectively. (**b**) The single 1330 or 1550 nm SLD has a spectral width of 80 nm. In both cases, for the dual-SLD demodulation, the 1330 nm SLD is with a 40 nm spectral width, and the 1550 nm SLD is with a 46 nm spectral width.

Under the same conditions, these fiber-optic F–P sensors were demodulated with only a single SLD. The relative deviations of the calculated cavity lengths obtained from the cross-correlation curve of a single SLD of 1330 or 1550 nm are shown in Figure 7a. It can be found that, especially for cavity lengths less than 100 µm, the demodulation deviations obtained by our proposed method are significantly smaller than those obtained using a single source. Therefore, the proposed dual SLD cross-correlation cavity length demodulation method can solve the problem of the main peak judgment ambiguity when the cavity length of the fiber-optic F–P sensor is too short or when the spectral width of the single light source used is too narrow. Using this method, fiber-optic F–P sensors with relatively short cavity lengths can be accurately demodulated.

The simulated demodulation results by the use of a single SLD with a wider bandwidth of 80 nm are given in Figure 7b. It can be found that, by using a single SLD with wider spectral range, the demodulation performances can be actually improved to some extent. Relative large errors caused by “mode jumping” that appeared for cavity lengths larger than 60 μm have been eliminated by the use of a wider bandwidth SLD. However, the demodulation performance is not as good as that based on two SLDs with different spectral ranges but a certain spectral separation. As seen from Figure 7b, for cavity lengths equal to or shorter than 60 μm, mode jumping problems appeared for both single 1330 and 1550 nm SLDs with an 8 nm bandwidth. However, the dual-SLD cross-correlation demodulation method can stably and reliably be used even for cavity lengths shorter than 15 μm, benefiting from its excellent “mode jumping” restrain ability.

## 4. Experimental Verification

**Figure 8:**
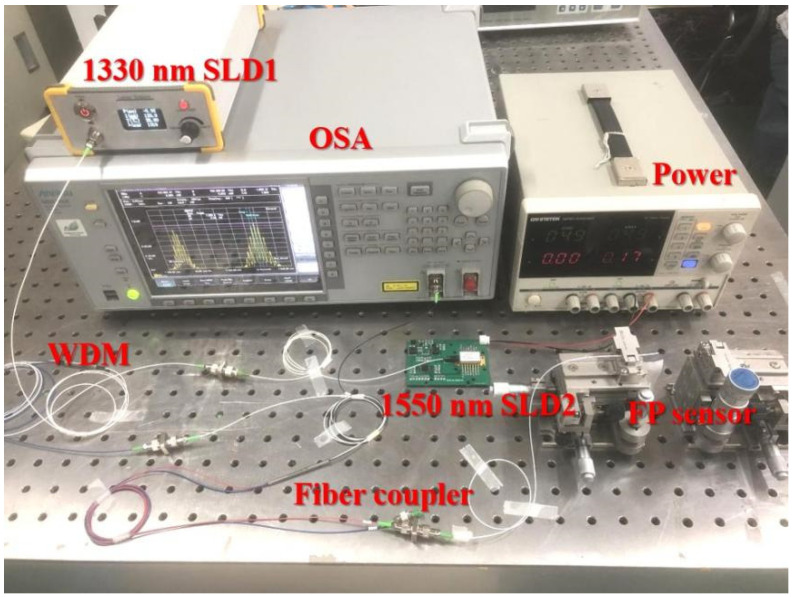
Experimental setup of the fiber-optic F–P sensor demodulation system.

**Figure 9:**
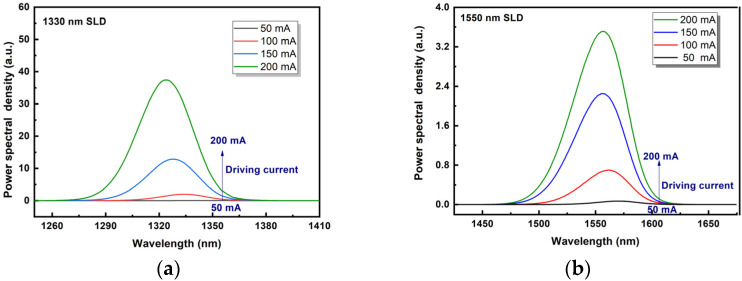
Output spectra of the two SLDs with central wavelengths of (**a**) 1330 nm and (**b**) 1550 nm under different driving currents.

To experimentally verify the proposed dual SLD cross-correlation demodulation method, a fiber-optic F–P sensor demodulation system was constructed, as shown in Figure 8. The system was composed of two SLDs, a 2 × 1 WDM, a 2 × 2 fiber coupler, and an OSA. The fiber-optic F–P sensor was directly connected to one port of the 2 × 2 fiber coupler. Output spectra of the two SLDs under different driving currents are shown in Figure 9. It can be noticed that the central wavelengths and 3-dB spectral ranges slightly vary with the changing of the driving current, and the output power is with a relatively large difference under the same driving current. To balance the output power, the driving current of SLD 1 is adjusted to be a relatively low value. The light beams emitted by SLD 1 (central wavelength: 1330 nm; linewidth: 40 nm) and SLD 2 (central wavelength: 1550 nm; linewidth: 46 nm) passed through the WDM and arrived at the 2 × 2 fiber coupler. The combined light beam was then coupled to the fiber-optic F–P sensor. The reflected light beam carrying the cavity length information was returned and finally received by the OSA (Anritsu, MS9740A, minimum wavelength resolution: 0.03 nm). All the fibers used in the system are standard communication SMFs (Corning SMF-28e), which can maintain single-mode and low-loss propagation for the two spectral bands we used. Subsequently, the optical spectrum extracted by the OSA was processed using a program written according to the proposed data processing algorithm to determine the real cavity length.

In these experiments, fiber-optic F–P sensors were fabricated by inserting two vertically cut SMFs into a glass capillary, similar to the structure shown in Figure 1. The detailed fabrication procedures are as follows: First, two SMFs are pealed off a short section of coating layer and are vertically cut. Second, with the help of a precision translation stage and under the monitoring of a microscope, one SMF is penetrated into a short section of glass capillary, which has an inner diameter approximate but is larger than the diameter of the SMF; then the SMF is fixed with the glass capillary by the use of a little ultraviolet (UV)-epoxy. Third, the other end of the SMF is connected to a broadband source, and the reflected light is monitored by an OSA. The second SMF is then penetrated into the glass capillary from the other end. With the monitoring of the reflection spectrum, the cavity length can be calculated to help tune the F–P cavity to any required length. Finally, after the F–P cavity is tuned to the required length, a little UV-epoxy is used to fix the second SMF to the glass capillary.

Sixteen fiber-optic F–P sensors with different standard cavity lengths in the range of 28–210 μm were manufactured and connected to the demodulation system individually. Their cavity lengths were 28.566, 40.124, 52.835, 64.493, 77.553, 8.693, 100.214, 111.358, 123.807, 137.518, 148.913, 159.233, 170.022, 183.873, 195.942, 207.965 μm, respectively. For the predetermination of these cavity lengths, the classical cross-correlation algorithm was used for the calculation, and an SLD with a relatively large spectral width (3-dB bandwidth of 90 nm) was used as the light source. Since the “mode jumping” phenomenon can be effectively avoided, the uncertainty of the predetermined cavity length was expected to be at a subnanometer level [26,27], which is sufficient in comparison with the proposed algorithm.

**Figure 10:**
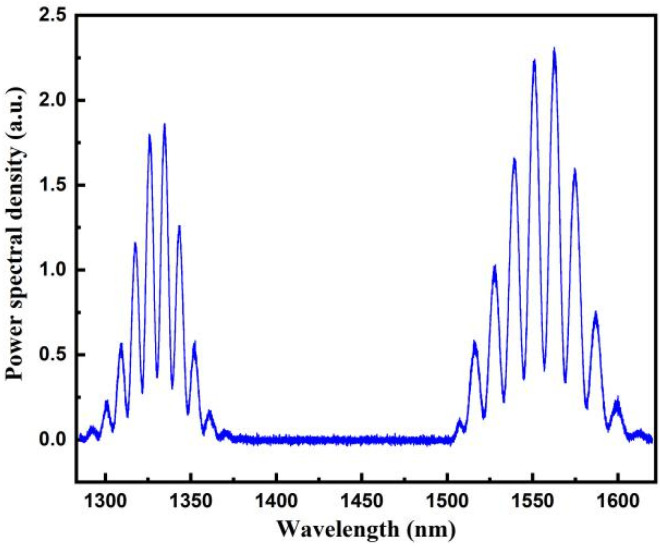
Reflection spectrum of a 100.214 μm fiber-optic F–P sensor.

**Figure 11:**
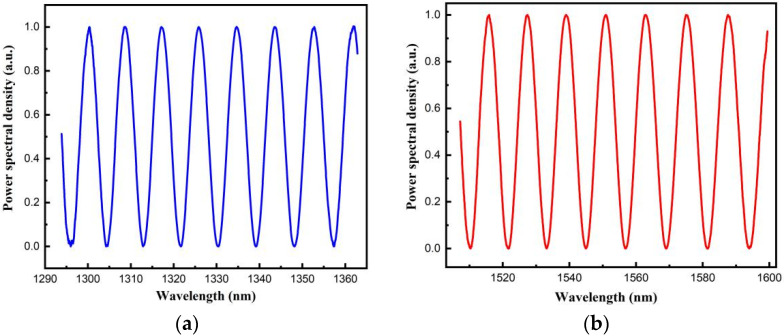
Normalized reflection spectra in the spectra ranges covered by (**a**) 1330 nm SLD and (**b**) 1550 nm SLD.

**Figure 12:**
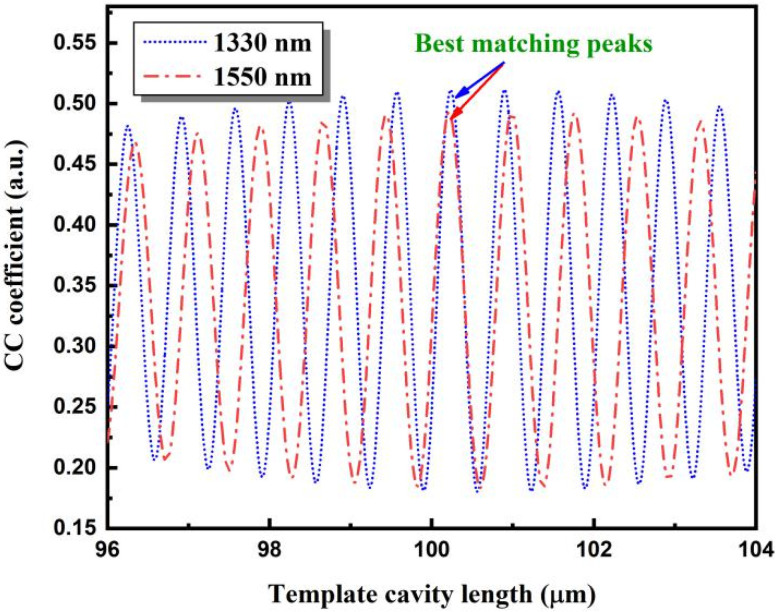
Cross-correlation curves taken from the reflection spectra of two different spectral ranges.

The reflection spectrum of the 100.214 μm fiber-optic F–P sensor is shown in Figure 10. The spectrum is treated using the proposed algorithm. The normalized spectral functions corresponding to the spectral ranges covered by the 1330 and 1550 nm SLDs are shown in Figure 11a,b, respectively. Cross-correlation calculations were performed for both spectral functions for a template cavity length range of 96–104 μm, and the two cross-correlation curves were superimposed, as shown in Figure 12. The best matching peaks were found to be near the template cavity length of 100 μm. Therefore, the main peaks of both cross-correlation curves were determined. Following a peak positioning process, and averaging the cavity lengths, the cavity length was calculated to be 100.218 μm, which deviates from the standard cavity length by 0.004 μm, and the relative deviation is 0.004%.

**Figure 13:**
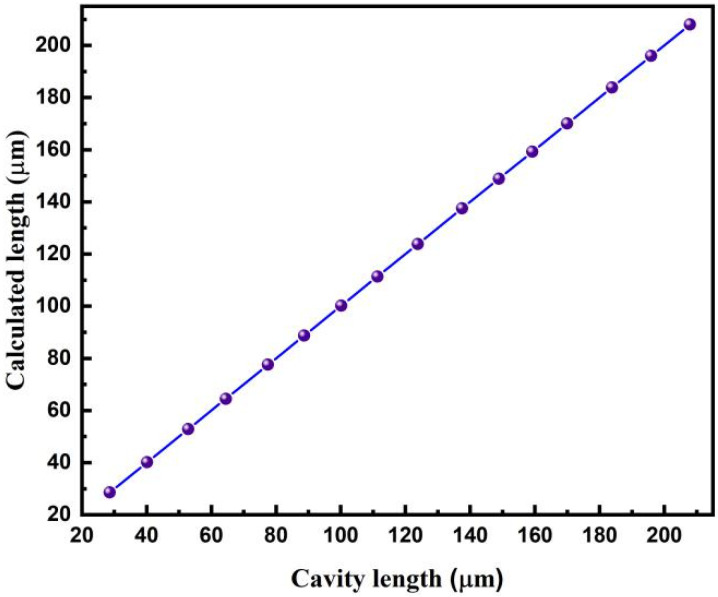
Relationship between the calculated cavity length and the real cavity length for fiber-optic F–P sensors through the proposed dual SLD cross-correlation demodulation method.

**Figure 14:**
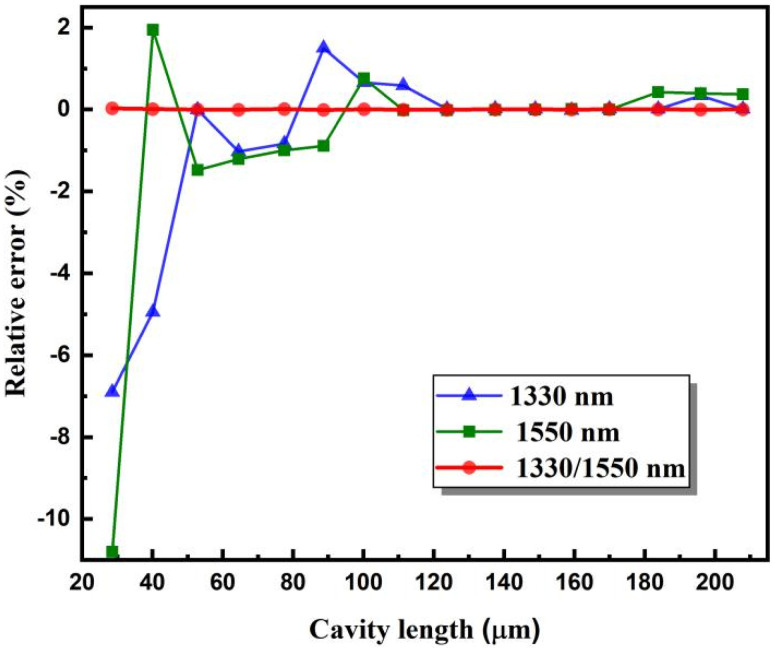
Demodulation deviations of fiber-optic F–P sensors with cavity lengths in the range of 20–200 μm under different illuminating conditions of a single 1330 or 1550 nm SLD or two SLDs simultaneously.

Other fiber-optic F–P sensors were demodulated using the same approach. The relationship between the demodulated cavity lengths and the real values is shown in Figure 13. A good linear relationship was observed. The maximum deviation was approximately 0.008 μm. The dual SLD cross-correlation demodulation results were also compared with the results obtained with only one SLD using the conventional cross-correlation algorithm. As shown in Figure 14, for the entire cavity length range, the proposed method maintains high accuracy. When only one SLD was used, large relative errors appeared when the cavity length was shorter than 100 μm. The advantages of the proposed dual SLD cross-correlation demodulation method over the conventional cross-correlation method are evident, especially when the cavity length is relatively short.

**Figure 15:**
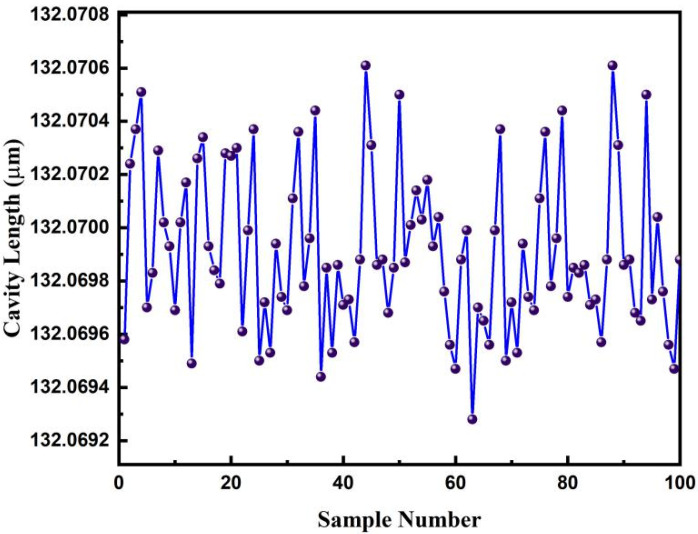
Sampling results from the continuous demodulation of a fiber-optic F–P sensor by the dual SLD cross-correlation demodulation method taken over 100 times.

The demodulation resolution of the proposed method was also investigated by repeatedly demodulating a fiber-optic F–P sensor with a fixed cavity length. The measurement results with 100 continuous samplings are shown in Figure 15. The maximum demodulated cavity length was 132.071 μm, and the minimum demodulated cavity length was 132.069 μm. The range of the measurement change was approximately 0.0013 μm, and the standard deviation of the measurement results was 0.0005 μm. Therefore, the proposed method achieved a demodulation resolution of 0.0005 μm.

## 5. Conclusions

In conclusion, a high-precision cross-correlation cavity length demodulation method based on dual SLDs with different central wavelengths for fiber-optic F–P sensors is proposed. This method can overcome the main peak judgment ambiguity of the cross-correlation curve, which may appear when the cavity length of the fiber-optic F–P sensor is too short or when the spectral width of the single light source used is too narrow. The proposed method can significantly improve the cavity length demodulation accuracy of fiber-optic F–P sensors under these conditions. Although an ultrabroadband light source, such as a supercontinuum source, may achieve similar or better results, the proposed method provides a cost-effective solution for the accurate demodulation of short-cavity-length F–P sensors. Fiber-optic F–P sensors with cavity lengths in the range of 20–200 μm were successfully demodulated in real experiments. The maximum deviation was found to be 0.008 μm, and the standard deviation of the measurement after 100 times of sampling was only 0.0005 μm. These results show that the dual SLD cross-correlation demodulation method can achieve high precision and good stability. Therefore, this method has the potential to be widely used in real applications of fiber-optic F–P sensors.

## Data Availability

Data underlying the results presented in this paper are not publicly available at this time but may be obtained from the authors upon reasonable request.
